# Difficulties and Problematic Steps in Teaching the Onstep Technique for Inguinal Hernia Repair, Results from a Focus Group Interview

**DOI:** 10.1155/2016/4787648

**Published:** 2016-04-10

**Authors:** Kristoffer Andresen, Jannie Laursen, Jacob Rosenberg

**Affiliations:** Department of Surgery, Herlev Hospital, University of Copenhagen, Herlev Ringvej 75, 2730 Herlev, Denmark

## Abstract

*Background*. When a new surgical technique is brought into a department, it is often experienced surgeons that learn it first and then pass it on to younger surgeons in training. This study seeks to clarify the problems and positive experiences when teaching and training surgeons in the Onstep technique for inguinal hernia repair, seen from the instructor's point of view.* Methods*. We designed a qualitative study using a focus group to allow participants to elaborate freely and facilitate a discussion. Participants were surgeons with extensive experience in performing the Onstep technique from Germany, UK, France, Belgium, Italy, Greece, and Sweden.* Results*. Four main themes were found, with one theme covering three subthemes:* instruction of others (experience, patient selection, and tailored teaching), comfort, concerns/fear, and anatomy*.* Conclusion*. Surgeons receiving a one-day training course should preferably have experience with other types of hernia repairs. If trainees are inexperienced, the training setup should be a traditional step-by-step programme. A training setup should consist of an explanation of the technique with emphasis on anatomy and difficult parts of the procedure and then a training day should follow. Surgeons teaching surgery can use these findings to improve their everyday practice.

## 1. Introduction

When a new surgical procedure is introduced, the common way for training surgeons is to assist and observe the procedure for a number of times and then progressively take over steps of the procedure, until they can perform the whole procedure by themselves [1]. This way of training is applicable when both experienced surgeons and surgeons in training work together for extended time and can perform the required number of procedures to train the inexperienced surgeons to an acceptable level. When a new technique is brought into a department, it is often experienced surgeons that learn it first and then they pass it on to the surgeons in training. Experienced surgeons have to move quickly from supervised training and into a self-training-level, since an instructor (of the new technique) cannot stay in the department for a long time.

The Onstep technique is a new technique for the repair of inguinal hernias [2, 3]. Since this technique is new, external instructors, often at a training centre, handle the training of surgeons who want to learn the technique. In other settings the instructor visits the surgeons' centre/clinic and the instruction/training is performed on site, the so-called proctoring [4].

The training schedule for the Onstep technique currently constitutes one evening with lecture and video demonstration followed by a day in the operating room. In the operating room the instructor performs the first procedure and the surgeons learning the new technique perform the following 4-5 procedures.

It seems safe to spend only one day of supervised instruction/training before the surgeons do self-training, as long as the trainees are surgeons with expertise in other hernia repair techniques [3]. Since this way of training does not give the experienced trainee opportunity to gradually take over the procedure, the gain of knowledge and technical skills from the short visit has to be as high as possible. The experience for the instructing surgeons has not yet been investigated, and difficulties with the transfer from supervised training to self-training have not been described.

This study seeks to clarify the issues and problems as well as positive experiences that arise when teaching and training surgeons in the Onstep technique, seen from the instructor's point of view.

## 2. Material and Methods

### 2.1. Design

Our approach and aim were to understand instructors' experience with the training of surgeons in the Onstep technique. Therefore, designing a qualitative study was decided, since quantitative methods do not allow participants to elaborate, discuss, and reach a higher understanding of the subject under investigation. A qualitative study using a focus group interview was designed, because this form of data collection allows participants to elaborate freely and share thoughts and facilitates discussion [5].

### 2.2. Participants

Eligible participants were surgeons with experience in performing the Onstep technique. The nine participants came from Germany, United Kingdom, France, Belgium, Italy, Greece, and Sweden. All participants had experience with hernia surgery in general and all were experienced with both performing and teaching the Onstep technique.

### 2.3. Data Collection

Before the focus group interview, a pilot interview was done, to ensure the quality of the interview-model and the open-ended questions [6]. The focus group interview took place without disturbances and all participants were encouraged to express themselves freely and allow for other opinions to be expressed.

### 2.4. Methodological Orientation and Theory

The focus group interview was chosen as the method for studying instructors' experience with the training of surgeons in the Onstep technique. In order to structure the focus group interview, an interview guide with open-ended questions was used (see Table 1) [7]. The interview guide was drafted by Kristoffer Andresen and Jannie Laursen. The interview guide was pilot tested and following results and feedback from the pilot test, the final version was reached through discussion among the authors. The research team constituted one professor of surgery, with extensive experience in teaching the Onstep technique and experience in overseeing focus group studies, one researcher whose primary methodological orientation is qualitative research, and one researcher who is currently overseeing studies of the Onstep technique.

### 2.5. Data Analysis

The interview was recorded and transcribed verbatim in order to allow full transparency in analysis. Data saturation was reached when no new approaches or new meanings came up during the interview. The approach to the interview was descriptive and reflective, in order to capture all meaningful elements of the surgeons' experiences. Two researchers (Kristoffer Andresen, Jannie Laursen) performed the analysis in parallel processes. The analysis and results were compared and discussed until consensus was reached [7].

The qualitative content analysis focused on both manifest and latent content. Manifest content is what was said explicitly and latent content was issues that were implied during the interview. The unit of analysis was the experience of instructing and teaching other surgeons in the Onstep technique. First the transcribed interview was read multiple times in order to obtain a sense of the whole. Then the text about the unit of analysis was extracted and the text was then divided into meaning units and condensed. Subsequently, the condensed meaning units were abstracted and labelled with a code [8]. When agreement was reached about the codes, the content was formulated into themes. We present and exemplify the results with citations from the interview.

### 2.6. Ethical and Data Safety Considerations

This study was approved by the Danish Data Protection Agency (Journal number: HEH-2013-040). The study did not need ethical approval from the Ethical Committee of the Capital Region of Denmark, but we obtained a statement of this from the ethics committee: H-2-2013-FSP55.

Before the interview was conducted, eligible participants were informed orally and received a written description of the project. In order to allow the participants to elaborate freely, they were assured that all recordings would be kept confidential, the inputs of all participants would be anonymous, and they were free to withdraw consent to participate at any time. No participants were paid for their participation in the interview.

The surgeons were encouraged to talk about and discuss their experience with learning, teaching, and performing the technique. Therefore, they would be talking about their own performance and possibly their colleagues' and students'. This highlights the need for keeping data confidential and therefore the country of residence and experience level of the individual participants are not stated.

In Results* trainee* refers to an experienced surgeon learning the Onstep technique if not otherwise stated.

## 3. Results

Four main themes were found, with one theme covering three subthemes:* instruction of others (experience, patient selection, and tailored teaching), comfort, concerns/fear, and anatomy; see Table 2.*

### 3.1. Instruction of Others

#### 3.1.1. Experience

When talking about instruction of others, the experience of the trainee was discussed. It was agreed that experience with surgery and especially hernia surgery made it easier to learn Onstep. Laparoscopic experience makes it easier for the trainee to understand the anatomy of the preperitoneal space that is not observed during the Lichtenstein technique. It was discussed that even experienced surgeons can have problems understanding how the Onstep technique is performed. The difficulties, in spite of experience, could be explained by the new way of entering the inguinal canal and operating in the preperitoneal space from an open approach. This is usually done with a laparoscopic approach.

#### 3.1.2. Patient Selection

The group agreed that it is important to choose the right patients when teaching the Onstep technique. Patients have to be slim, with a primary, small, and not scrotal hernia. Patients that fulfil these criteria will be better suited for demonstration of the technique because it is easier to visualize the anatomy and handle the hernia. If the patient is obese or has a big, difficult hernia, the trainees and the trainer will have some concerns during the operation whether the procedure can be performed. On the other hand, the benefit of a difficult case during a training day is to show the trainees that the Onstep can be performed, thereby lending credit to the technique.

#### 3.1.3. Limitations/Tailored Teaching

Participants agreed that the teaching has to be tailored to the needs of the trainees. Especially with inexperienced trainees, there is a need to focus on limitations and challenges that can arise during an Onstep repair. If the limitations and difficult steps are omitted during the teaching, the trainees might not be aware of them and perform the Onstep inappropriately, that is, on patients with hernias that are not easily manageable by the Onstep technique such as scrotal or very large hernias. It is essential to point out the anatomy to inexperienced trainees to make them understand the procedure in relation to the anatomy. This might be especially true for inexperienced surgeons, but even for the experienced surgeons there is a need to demonstrate the anatomy and landmarks.

### 3.2. Comfort

Both the trainer and trainee can be under stress during training. The trainer can be under stress if the hernia is difficult or the patient is obese and it is difficult to demonstrate the technique. This stress can be avoided or minimized with proper patient selection. The trainers either received trainees at their own department or went to other departments for training. Trainers that had experienced the latter said that it increased their stress but that the trainees were more in their comfort zone. Furthermore, the staff being present at the operation would also see the Onstep technique. The anaesthetist could see what should be used and the scrub nurse could be convinced that the Onstep technique is a good technique. The trainees are more in their comfort zone when they are being trained at their own department since they know the staff, the routines, and the equipment.

In order to make the trainees feel comfortable with the technique, they have to understand the anatomy. When the trainees have seen and understood where the different landmarks of the anatomy are located when performing the Onstep technique, they are more comfortable and more willing to accept the technique.

### 3.3. Concerns/Fears

The trainers were concerned about two things: their own reputation as surgeons or teachers and the reputation of the technique. The underlying concern was complications from the technique. The trainers were concerned that if their trainees were trained poorly they might perform the Onstep technique wrongly and the reputation of the technique and their reputation as teachers could be damaged.

The trainers discussed what they saw as the concerns from the trainees. They had experienced that trainees were concerned about the preperitoneal space and especially the iliac vessels. This concern made them uncomfortable when conducting the steps of the Onstep technique that involves the preperitoneal space. One way to overcome this concern is to demonstrate exactly where the vessels are located during the operation thereby making it easy for the trainee to avoid them.

### 3.4. Anatomy

Knowledge and understanding of the anatomy in the inguinal area were mentioned and discussed in relation to the above-mentioned themes. One way to help the trainees understand the anatomy and enhance their orientation in the operative field would be to identify certain structures or landmarks. This should be done on every training occasion and for the trainees for every subsequent procedure they perform. By doing so, the trainees and trainer will at all times throughout the procedure know where they are operating and where they are placing the mesh. When the trainees are made aware of the anatomy and understand it, their concerns about placing a mesh in a new area will be minimized. A good anatomical understanding in relation to the technique will also increase the acceptance of the technique.

The trainers had experienced that trainees with experience from both laparoscopic and Lichtenstein repair tended to have a better understanding of the anatomy. Such experience makes it easier to learn the technique since learning the Onstep technique requires a good anatomical orientation.

## 4. Discussion

We found that experienced surgeons that performed instruction of others in the Onstep technique were discussing four main themes in relation to the subject:* anatomy, comfort, concerns, and instruction of others*. “Instruction of others” could further be divided into* experience*,* patient selection,* and* tailored teaching*. The trainers agreed that experience with surgery in general and specifically hernia repair is a must for trainees to learn enough at a one-day training session to start training by themselves. Even though the trainers were all experienced hernia surgeons, they themselves had difficulties grasping the way Onstep was performed, based only on lectures and drawings. However, they understood and could perform the technique after they had seen and tried it. This highlights the need to integrate hands-on training in the training programme.

With regard to patient selection, the trainers found that the patient and the hernia had to be suitable for training, both because a difficult case could scare some trainees from the method, if it seemed too difficult, and because a difficult case would be stressful for the teacher. Therefore, trainers have to make sure they are well aware of which patients are scheduled on the day of training. On the other hand, a difficult case might be used in order to demonstrate that the technique can work, even on difficult cases. A certain amount of stress related to challenges experienced has been found to have a positive effect on learning [9]. Thereby a difficult case that is challenging but not impossible might be very helpful for demonstration and learning during the training day.

Tailoring of teaching had to be done to accommodate the different learners that the trainers met. For surgeons in training, with limited or no experience in hernia surgery, a step-by-step approach was advised, as opposed to experienced trainees that would know how and to which patients the Onstep could or should be applied. This indicates that surgeons with different levels of experience should not attend the same training sessions, especially when time is limited.

The comfort, for both the trainer and the trainee, was thought to affect the learning outcome of a training session. The trainers were willing to accept a higher level of discomfort, by doing local proctoring (training at the facility of the trainees) as a trade-off for having the trainees being more comfortable during training. The local proctoring allows the trainee to be more in the comfort zone, when learning the new technique [4]. When learners are in their comfort zone, the level of stress is diminished. Previous studies have found that higher levels of experienced stress can be a hindrance for learning [9], which could support the use of local proctoring in teaching the Onstep technique. Furthermore, the continued use of the method could be facilitated by local proctoring. Thereby the transfer from performing the procedure under supervision to unsupervised training should be easier, because the assisting staff already know the procedure [4]. When the trainee is less stressed during training, it becomes easier for them to focus on learning the procedure. Thereby lower level of stress in itself can also increase the learning outcome [10], as long as the local proctoring does not put too much stress on the trainer.

When discussing a surgical procedure, the theme of anatomy naturally arises. The anatomy of the inguinal canal is in the core curriculum of all medical students, but the practical aspect of entering and identifying anatomical landmarks in the inguinal canal during a hernia repair is difficult. Live demonstration of a surgical procedure has been found to reinforce the learners understanding and knowledge of a surgical technique [11].

Some studies have investigated medical students' learning in the OR and found that being an active part of the procedure by assisting the surgeon increased the learning [12]. Although learners in our study were far beyond their time as medical students, the setup of training is focused around hands-on training, which will enhance learning for this type of learners for the Onstep procedure.

This study was done on a single group of surgeons, which could be seen as a limitation. However, the group consisted of surgeons from seven different countries and we believe they were a valid representation of surgeons performing the Onstep procedure. Furthermore, the mix of different nationalities and cultural backgrounds makes these findings valid in an international perspective. The strengths of the study were that two researchers were present during the focus group interview [7], one that facilitated the discussion and one that ensured that important themes arising during the interview were followed, and that all participants were strongly encouraged to participate in the discussion. Furthermore, a test-interview was done beforehand in order to both train the researchers and test the questions in the interview guide.

Interestingly, the issue of the limited time the trainers spent with the trainees did not arise as a theme. This is probably because the time is sufficient for training the surgeons and therefore was not considered an issue. The instructors are not far away from their own learning situation (learning the Onstep technique), which is probably helping them in instructing others and thereby making them able to teach the procedure in a single training day [10].

These findings could be used to guide and inform everyday practice in surgical departments. If trainers as well as trainees are aware of difficulties and problematic steps when teaching/learning surgical techniques, they can develop new training programs or optimize current ones. When a surgeon in training and a trainer are only together for one evening and one day, the content should be as optimal as possible. For the evening lecture there should be a focus on anatomy in relation to the incision and placing of the mesh. Furthermore, it should be considered to discuss the difficult parts of the procedure and highlight to trainees that the difficulties exist. On the training day it is important to ensure the trainees are in their comfort zone. This can be done by proctoring in their departments and by carefully selecting the patients for the training programme. A structured feedback session should be performed after each procedure [13].

## 5. Conclusion

In conclusion, trainers of the Onstep technique found experience, patient selection, anatomy, and tailored teaching to be important when instructing others. A training setup should consist of a thorough explanation of the technique with emphasis on the anatomy and the difficult parts of the procedure and hands-on training day where all parts of the procedure as well as the anatomical landmarks are explained. Surgeons teaching surgery can use these findings to improve their everyday practice.

## Figures and Tables

**Table 1:** Interview guide.

Theme	Main question	Helping questions?
Opening	Tell me about the experience of training others in the Onstep technique	[An opening question to start the interview]

Instructions of others	What are your experiences when instructing/teaching the Onstep technique?	How do you organize your training sessions? How is the atmosphere/do you seek a certain atmosphere? What setup works well, do you or would you like to add something? Group dynamics, that is who could/should be trained together?

Compared to other techniques?	What are the main differences when you are instructing/teaching this technique?	Can anything from Lichtenstein be helpful? Anything from laparoscopy?

Which surgeons	Who should be taught this technique?	What prerequisites are preferred? That is: Experience, knowledge?
	Who could/should teach/train others?	Who can easily/hardly transfer from supervised training to self-training? Experience with other types of groin hernia surgery?

Proficiency	When is your student ready for unsupervised procedures?	What are the indicators for proficiency? (For yourself, for your students)? How could a tool be helpful? What is your experience with feedback? (Forms, structured feedback, focus on certain steps)?

Closure		Something else we need to add?

**Table 2:** Identified themes with examples from the interview.

Theme	Examples (citations)
Instruction of others	
Experience	With regard to the trainees - if they have already endoscopic procedure or endoscopic experience or if they have an experience in the preperitoneal space they learn much easier because they know this anatomy. The learning curve is none if you know how to perform a good inguinal hernia, anatomical dissection, and the technique, you can learn it very fast We thought we understood it (…), but when we saw it the next day it was completely different from what they explained to us the night before. Yet we were all reasonable experienced in hernia surgery. So there is a failure here to grasp the technique in many cases. If you don't get it right what they say, then people will do it wrong.
Patient selection	For the training procedure you should focus on – what is the best (type of patient) to start, it is important, because if you have a very tricky case at the beginning you will stop doing this (Onstep).
Tailored teaching	There is a difference (between experienced and inexperienced trainees) if you are just a trainee (…) it is a step by step program. The learning curve is none if you know how to perform a good inguinal hernia, anatomical dissection, and the technique, you can learn it very fast, but we have 16 trainees now in the department and many of them are good surgeons in performing ONSTEP technique, so it depends totally on the personal oriented technique.

Comfort	I think you must do everything, so that the trainee or the surgeon you have in your operating room, is feeling comfortable, otherwise anything that you will do won't help or will not succeed – he must feel comfortable during all the training and teaching. If I go to another place, they were happier, because they were using their own anaesthetist, they had their own theatre staff, their own senior staff - so it made me slightly more stressed – but they learned more because they were in their own comfort zone. Start with a simple case and when you feel comfortable – then you will stay on to the technique, otherwise you will leave it.

Concerns/fear	The fear for me was the vessels – that was the major fear and that what's everybody ask or are looking for when doing it, but if you overpass this fear I think that it is very easy and very comfortable to put your mesh on the hernia. It is very important that you don't destroy the reputation of ONSTEP in the beginning by teaching it the wrong way. They never do that (perform the technique un supervised) because that would ruin the technique and perhaps the reputation, not only of the technique but my reputation also.

Anatomy	With regard to the trainees - if they have already endoscopic procedure or endoscopic experience or if they have an experience in the preperitoneal space they learn much easier because they know this anatomy. Of course they normally are very surprised of the small incision and to look inside the preperitoneal space and we show them also to look inside, to look for the Cooper ligament, the cord and anything else, so they learn very fast, if they have the experience. (…) they are afraid to make some damage to the vessels, maybe because the anatomy of this part is not really clear to all surgeons. So, I think that one of the biggest problems is the anatomy. It has always been the anatomy and it still is.
